
JOURNAL INFORMATION
==============================
NLM Title Abbreviation: ScientificWorldJournal
Journal ID: ScientificWorldJournal
NLM Journal ID: 101131163
Journal ID: TSWJ

The Scientific World Journal

ISSN: 2356-6140
EISSN: 1537-744X
Publisher: Wiley

ARTICLE INFORMATION
==============================
PMCID: PMC5848720
PMID: 18246282
DOI: 10.1100/tsw.2008.21
Article ID: 960658
Article version: 1
Subjects: Editorial

Evaluation of Project P.A.T.H.S. in Hong Kong: Triangulation of Findings Based on Different Evaluation Strategies

Shek Daniel T. L. 1 2 3 *
1 Centre for Quality of Life, Hong Kong Institute of Asia-Pacific Studies, The Chinese University of Hong Kong, Hong Kong
2 Social Welfare Practice and Research Centre, The Chinese University of Hong Kong, Hong Kong
3 Kiang Wu Nursing College of Macau, Macau
*Daniel T. L. Shek: danielshek@cuhk.edu.hkAcademic Editor: Joav Merrick

Publication date: 2008
Electronic publication date: 2008 Jan 14
Volume: 8
First page: 1
Last page: 3
Received 2007 Nov 15; Revised 2007 Nov 26; Accepted 2007 Nov 28
Copyright: Copyright © 2008 Daniel T. L. Shek.
Copyright year: 2008
License: This is an open access article distributed under the Creative Commons Attribution License, which permits unrestricted use, distribution, and reproduction in any medium, provided the original work is properly cited.
License URL: https://creativecommons.org/licenses/by/3.0/

Funding: Hong Kong Jockey Club Charities Trust

==============================
Editorial
Special Issue: Evaluation of Project P.A.T.H.S. in Hong Kong
*TheScientificWorldJOURNAL* (2008) 8, 1–3
TSW Holistic Health & Medicine
ISSN 1537-744X; DOI 10.1100/tsw.2008.21

TheScientificWorldJOURNAL
www.thescientificworld.com

### Evaluation of Project P.A.T.H.S. in Hong Kong: Triangulation of Findings Based on Different Evaluation Strategies

Daniel T.L. Shek[1,2,3]

*[1]Centre for Quality of Life, Hong Kong Institute of Asia-Pacific Studies, The Chinese University of Hong Kong; [2]Social Welfare Practice and Research Centre, The Chinese University of Hong Kong; [3]Kiang Wu Nursing College of Macau, Macau*

**E-mail**: danielshek@cuhk.edu.hk

*Received November 15, 2007; Revised November 26, 2007; Accepted November 28, 2007; Published January 14, 2008*

---

There are different adolescent developmental issues in Hong Kong, such as mental health problems, abuse of psychotropic substances, adolescent suicide, school violence, and drop in family solidarity, that deserve the attention of helping professionals[1]. With reference to these problems, primary prevention programs that target specific adolescent developmental problems and positive youth development programs are called for. Furthermore, attempts to identify the "at-risk" adolescents at an earlier stage are important. However, research findings show that there are very few systematic and multiyear positive youth development programs in Hong Kong. Even if such programs exist, they commonly deal with isolated problems and issues in adolescent development (i.e., deficits-oriented programs) and they are relatively short term in nature. In addition, systematic and long-term evaluation of the available programs does not exist[2].

To promote holistic development among adolescents in Hong Kong, the Hong Kong Jockey Club Charities Trust approved HK$400 million to launch a project entitled "P.A.T.H.S. to Adulthood: A Jockey Club Youth Enhancement Scheme". "P.A.T.H.S." denotes Positive Adolescent Training through Holistic Social Programmes. The Trust invited academics of five universities in Hong Kong to form a research team, with The Chinese University of Hong Kong as the lead institution, to develop a multiyear universal positive youth development program to promote holistic adolescent development in Hong Kong. Besides developing the program, the research team also provides training for teachers and social workers who implement the program and carry out longitudinal evaluation of the project[3].

There are two tiers of programs (Tier 1 and Tier 2) in this project. The Tier 1 Program is a universal positive youth development program where students in Secondary 1 to 3 participate in the program, normally with 20 h of training in the school year at each grade. Because research findings suggest that roughly one-fifth of adolescents would need help of a deeper nature, the Tier 2 Program is generally provided for at least one-fifth of the students who have greater psychosocial needs at each grade (i.e., selective program). To promote the positive development of adolescents, a total of 15 adolescent developmental constructs are covered in the project, particularly the Tier 1 Program. These include: promotion of bonding, cultivation of resilience, promotion of social competence, promotion of emotional competence, promotion of cognitive competence, promotion of behavioral competence, promotion of moral competence, cultivation of self-determination, promotion of spirituality, development of self-efficacy, development of a clear and positive identity, promotion of beliefs in the future, provision of recognition for positive behavior, provision of opportunities for prosocial involvement, and fostering prosocial norms.

There are two implementation phases in this project – Experimental Implementation Phase and Full Implementation Phase. For the Experimental Implementation Phase of the Secondary 1 program (January 2006 to August 2006), 52 secondary schools participated with the objectives of accumulating experience in program implementation and familiarizing frontline workers with the program design and philosophy. In the

©2008 with author.
Published by TheScientificWorld; www.thescientificworld.com

2006/07 school year, the programs were implemented on a full scale at the Secondary 1 level. In the 2007/08 school year, the programs were implemented at Secondary 1 and 2 levels. In the 2008/09 school year, the programs will be implemented at Secondary 1, 2, and 3 levels.

The project is pioneering and groundbreaking in Hong Kong and other Chinese contexts in many aspects. First, the full Tier 1 Program provides 60 h of universal training on positive youth development in the junior secondary school years. Second, it includes both universal and selective programs (i.e., primary and secondary prevention programs). Third, it includes the development of a multiyear positive youth development program, provision of training on a massive scale, and implementation of rigorous evaluation. Fourth, it involves the collaboration of five universities, different government departments, NGOs providing school social work service, and the education sector. Fifth, it has been earmarked with a record high amount of grant. Finally, it utilizes longitudinal evaluation over a long period of time involving the collection of different data from different sources.

Although there are many unique attributes of the project, the usefulness of the project remains to be demonstrated. To provide a comprehensive picture of the effectiveness of the project, several evaluation strategies are employed, including objective outcome evaluation, subjective outcome evaluation, qualitative evaluation based on focus groups, student diaries and in-depth interviews, process evaluation, and interim evaluation. To date, there are research findings that have demonstrated the effectiveness of the program via objective outcome evaluation[4], subjective outcome evaluation[5,6,7,8], process evaluation[9], interim evaluation[10], qualitative evaluation[11], and convergence of subjective outcome evaluation and objective outcome evaluation findings[12,13].

To further accumulate empirical evidence on the effectiveness of the program, this special issue is a collection of several additional papers on the evaluation of the Tier 1 Program. First, based on an experimental design, Shek, Siu, Lee, Cheung, and Chung reported that the experimental group performed better than the control group on different measures of positive youth development based on the data collected in the first year of the Full Implementation Phase. Second, qualitative evaluation strategies were used to examine the experiences of the program participants and program implementers. In the paper by Shek, Sun, Lam, Lung, and Lo, Secondary 1 students participating in Year 1 of the Experimental Implementation Phase were invited to write a reflective journal in the form of weekly diary to reveal their perceptions and feelings regarding the Tier 1 Program and the related benefits after completion of the program. Results generally showed that the respondents had positive views of the program as well as the instructors, and they perceived benefits of joining the program. In the next paper, Shek carried out secondary data analyses based on the conclusions drawn by the workers in the 52 evaluation reports submitted by the participating schools. Generally speaking, results showed that most of the conclusions concerning perceptions of the Tier 1 Program, instructors, and effectiveness of the programs were positive in nature, although responses on the difficulties encountered and suggestions for improvements were observed.

Third, to understand the nature of program implementation, process evaluation was carried out. In the paper by Shek, Ma, Sun, and Lung, implementation quality of the Tier 1 Program (Secondary 1 Curriculum) of the Project P.A.T.H.S. was examined. Consistent with the previous findings, results showed that the overall level of program adherence was generally high and high implementation quality of the program was found. In the paper by Shek, Lee, and Sun documenting process evaluation of the Tier 1 Program (Secondary 2 Curriculum), the findings similarly showed that the implementation quality was high at the Secondary 2 level.

Fourth, to gain a broad view of the implementation quality of the programs, interim evaluation was carried out. Shek, Ma, and Sun reported findings based on the interim evaluation of the Tier 1 Program (Secondary 1 Curriculum). Results showed that most workers perceived that the students had positive responses to the program and the program was beneficial to the students, although negative comments on the program design and difficulties in the implementation process were also recorded. In the next paper, Shek, Sun, and Siu examined the implementation quality of the Tier 1 Program (Secondary 2 Curriculum). Consistent with the previous findings, results showed that the program was well received by the program participants and implementers, although some difficulties were expressed.

Finally, based on subjective outcome evaluation, Shek, Lee, Sun, and Lung examined the nature of the Tier 2 Program based on the responses of 2,173 students in 52 schools. Results showed that high proportions of the respondents had positive perceptions of the programs, and the instructors and roughly four-fifths of the respondents regarded the program as helpful to them.

The above papers generally suggest that the Project P.A.T.H.S. was positively perceived by the program participants and implementers. Utilizing the principle of triangulation, an integration of the findings generated from different evaluation strategies revealed the positive nature of the Project P.A.T.H.S. It is hoped that through accumulation of evaluation findings related to Project P.A.T.H.S., effects of the project can be further understood. In view of the fact that evaluation findings on positive youth development in the Chinese culture are almost nonexistent[14], the present findings can be regarded as interesting and pioneering additions to the literature.

### ACKNOWLEDGMENTS

The preparation for this paper and Project P.A.T.H.S. were financially supported by the Hong Kong Jockey Club Charities Trust.

### REFERENCES

1. Shek, D.T.L. (2006) Adolescent developmental issues in Hong Kong: relevance to positive youth development programs in Hong Kong. *Int. J. Adolesc. Med. Health* **18(3),** 341–354.
2. Shek, D.T.L. (2006) Conceptual framework underlying the development of a positive youth development program in Hong Kong. *Int. J. Adolesc. Med. Health* **18(3),** 303–314.
3. Shek, D.T.L. and Ma, H.K. (2006) Design of a positive youth development program in Hong Kong. *Int. J. Adolesc. Med. Health* **18(3),** 315–327.
4. Shek, D.T.L. (2006) Effectiveness of the Tier 1 Program of the Project P.A.T.H.S.: preliminary objective and subjective outcome evaluation findings. *TheScientificWorldJOURNAL* **6,** 1466–1474. DOI 10.1100/tsw.2006.238.
5. Shek, D.T.L. and Ma, H.K. (2007) Subjective outcome evaluation of the Project P.A.T.H.S.: findings based on the perspective of the program participants. *TheScientificWorldJOURNAL* **7,** 47–55. DOI 10.1100/tsw.2007.38.
6. Shek, D.T.L., Siu, A.M.H., and Lee, T.Y. (2007) Subjective outcome evaluation of the Project P.A.T.H.S.: findings based on the perspective of the program implementers. *TheScientificWorldJOURNAL* **7,** 195–203. DOI 10.1100/tsw.2007.43.
7. Shek, D.T.L. and Sun, R.C.F. (2007) Subjective outcome evaluation of the Project P.A.T.H.S.: qualitative findings based on the experiences of program participants. *TheScientificWorldJOURNAL* **7,** 686–697. DOI 10.1100/tsw.2007.126.
8. Shek, D.T.L. and Sun, R.C.F. (2007) Subjective outcome evaluation of the Project P.A.T.H.S.: qualitative findings based on the experiences of program implementers. *TheScientificWorldJOURNAL* **7,** 1024–1035. DOI 10.1100/tsw.2007.161.
9. Shek, D.T.L., Ma, H.K., Lui, J.H.Y., and Lung, D.W.M. (2006) Process evaluation of the Tier 1 Program of the Project P.A.T.H.S. *TSW Holistic Health & Medicine* **1,** 300–309. DOI 10.1100/tswhhm.2006.240.
10. Shek, D.T.L. and Sun, R.C.F. (2006) Implementation of the Tier 1 Program of the Project P.A.T.H.S.: interim evaluation findings. *TheScientificWorldJOURNAL* **6,** 2274–2284.DOI 10.1100/tsw.2006.356.
11. Shek, D.T.L., Lee, T.Y., Siu, A., and Lam, C.M. (2006) Qualitative evaluation of the Project P.A.T.H.S. based on the perceptions of the program participants. *TSW Holistic Health & Medicine* **1,** 290–299. DOI 10.1100/tswhhm.2006.239.
12. Shek, D.T.L., Lee, T.Y., Siu, A.M.H., and Ma, H.K. (2007) Convergence of subjective outcome and objective outcome evaluation findings: insights based on the Project P.A.T.H.S. *TheScientificWorldJOURNAL* **7,** 258–267. DOI 10.1100/tsw.2007.59.
13. Shek, D.T.L. and Sun, R.C.F. (In press) Linkage between subjective outcome evaluation findings and objective outcome evaluation findings: a pioneer study in the Chinese culture. In *Educational Evaluation: 21st Century Issues and Challenges*. Columbus, F., Ed. Nova Science Publishers, New York.
14. Shek, D.T.L., Chan, Y.K., and Lee, P.S.N. (2005) Quality of life in the global context: a Chinese response. *Soc. Indicat. Res*. 71, 1–10.

**This article should be cited as follows:**

Shek, D.T.L. (2008) Evaluation of Project P.A.T.H.S. in Hong Kong: triangulation of findings based on different evaluation strategies. *TheScientificWorldJOURNAL:* TSW Holistic Health & Medicine **8**, 1–3. DOI 10.1100/tsw.2008.21.
